# Hereditary multiple exostoses: an educational review

**DOI:** 10.1186/s13244-025-01899-6

**Published:** 2025-02-21

**Authors:** Alvaro Rueda-de-Eusebio, Sara Gomez-Pena, María José Moreno-Casado, Gloria Marquina, Juan Arrazola, Ana María Crespo-Rodríguez

**Affiliations:** 1 Department of Radiology, Hospital Clínico San Carlos, Madrid, Spain; 2 Biomedical Imaging Research Group, Instituto de Investigación Sanitaria San Carlos (IdISSC), Madrid, Spain; 3 EURACAN Referral Centre, Madrid, Spain; 4 Department of Medical Oncology, Hospital Clínico San Carlos, Madrid, Spain; 5 Department of Medicine, School of Medicine, Universidad Complutense de Madrid (UCM), Madrid, Spain; 6 Instituto de Investigación Sanitaria San Carlos (IdISSC), Madrid, Spain; 7 Department of Radiology and Rehabilitation, School of Medicine, Universidad Complutense de Madrid (UCM), Madrid, Spain

**Keywords:** Hereditary multiple exostoses, Osteochondroma, Conventional radiography, Magnetic resonance imaging, Chondrosarcoma

## Abstract

**Abstract:**

Hereditary multiple exostoses (HME), an autosomal dominant disorder with an incidence of 1:50,000 to 1:100,000, is characterised by the formation of multiple osteochondromas arising from the metaphyses of long and flat bones. These osteochondromas often present as painless palpable lumps, though some cases are symptomatic due to mechanical compression or bursitis. Diagnosis of HME is typically clinical and radiological. WHO diagnostic criteria include ≥ 2 radiological osteochondromas in the juxta-epiphyseal region of the long bones. Genetic testing is reserved for ambiguous cases. HME is associated with mutations in the EXT-1 (exostosin-1) and EXT-2 (exostosin-2) genes. Imaging techniques, including conventional radiography, CT, MRI, ultrasound, and nuclear medicine, play a crucial role in diagnosing and assessing HME, with each modality offering distinct advantages in visualising the lesions and associated complications. Common complications include skeletal deformities, fractures, bursitis, as well as neural and vascular abnormalities. Notably, there is a 10% risk of malignant transformation into secondary chondrosarcoma in HME patients, compared to only a 1% risk in those with solitary osteochondromas. Malignant transformation should be suspected in patients with new-onset pain or specific imaging features in an osteochondroma, such as growth of de cartilaginous cap. In these cases, an MRI should be performed to assess the cartilage cap thickness. Advances in imaging techniques and genetic understanding have improved the management and prognosis of HME. Follow-up is essential to rule out malignant transformation. This review summarises current knowledge on the clinical presentation, pathogenesis, imaging characteristics, complications, and treatment of HME.

**Critical relevance statement:**

HME is a disorder characterised by the formation of osteochondromas arising from long and flat bones. Multi-modality imaging characteristics, clinical presentation, complications, and treatment are highlighted to familiarise the readers with this entity and offer optimal patient care.

**Key Points:**

- HME is characterised by multiple osteochondromas on long and flat bones.
- Imaging for HME includes radiography, CT, MRI, ultrasound, and nuclear medicine studies.
- Complications include non-malignant complications, such as bone deformities and malignant transformation.
- Cartilage-cap measurement with MRI or US is key to exclude malignancy.
- Follow-up is essential to rule out malignant transformation of the osteochondromas.

**Graphical Abstract:**

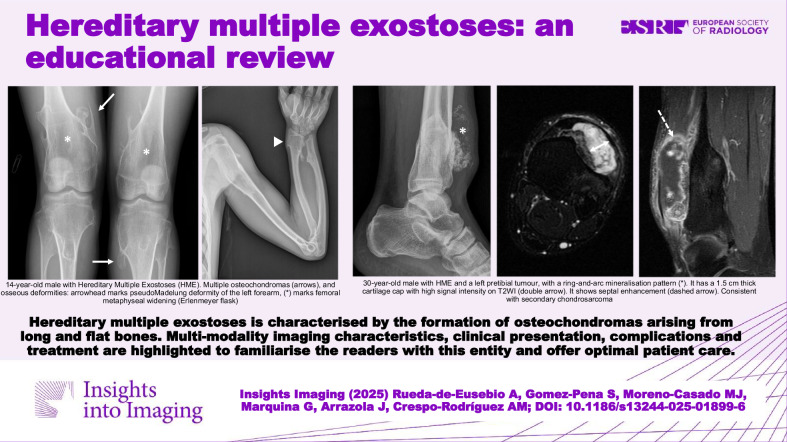

## Introduction

Hereditary multiple exostoses (HME), also known as hereditary multiple osteochondromatosis, hereditary deforming dyschondroplasia, diaphyseal aclasis, or multiple cartilaginous exostoses [1] has an incidence of 1:50,000–1:100,000 [2–4]. The disease is characterised by the development of multiple osteochondromas arising from the metaphyses of long and flat bones [5–7].

Osteochondromas are the most common bone neoplasms in children and adolescents, constituting 10–15% of all bone tumours and 20–50% of benign bone neoplasms [1]. The precise incidence of solitary osteochondromas remains uncertain, as many are asymptomatic and thus evade diagnosis. Nevertheless, their incidence is believed to be up to six times higher than that of HME [8].

Osteochondromas are hyaline cartilage-covered exostoses primarily originating from the metaphyses of long and flat bones, developing through endochondral ossification [5]. They vary in size and typically present a pathognomonic feature: a bony stalk with a medullary cavity that extends into the underlying bone [8].

The diagnosis of HME is suspected if more than two osteochondromas are detected, although most patients exhibit more than six lesions upon presentation, often accompanied by multifocal skeletal deformities [9, 10]. Diagnosis can be established based solely on clinical and radiological findings if multiple osteochondromas are found in the context of a compatible family history. Genetic testing may be conducted in order to identify a pathogenic genetic mutation if radiological findings or clinical history are inconclusive [5].

In recent years, progress has been made in various aspects of this disease, both in terms of genetic diagnosis and imaging assessment. The purpose of this article is to review the updated findings regarding clinical presentation, pathogenesis, imaging findings, complications, treatment and prognosis of patients with HME, illustrating the findings with cases from the archive of our Sarcoma Reference Centre.

## Clinical presentation

HME has a predilection for males (3:1 ratio) [1] and Caucasians [6]. Newborns are usually asymptomatic, while 50% of patients have a visible tumour by 5 years of age, and 80% by 10 years [2, 6]. The average age at diagnosis is 3 years [2].

As for osteochondromas, patients most often remain asymptomatic, and lesions are detected incidentally on radiographs obtained for other reasons, such as trauma [6, 11]. In symptomatic cases, the most common presentation is as painless palpable lumps, which may cause cosmetic problems [12, 13]. In some patients, the lesions may cause pain, mainly due to mechanical compression of adjacent structures such as nerves or blood vessels [5]. Bursitis formation around lesions, fracture of lesions and malignant transformation can also be painful [14].

There is variability in the size and number of exostoses, with a mean number of six lesions per patient [3]. The distribution of lesions throughout the skeleton is variable, with cases ranging from bilateral symmetrical disease to unilateral predominance, which may be related to genotype [15]. Lesions will tend to grow as long as the physics remains open, at a similar rate to the rest of the skeleton, and will stop increasing in size at skeletal maturity [1].

**Fig. 1:**
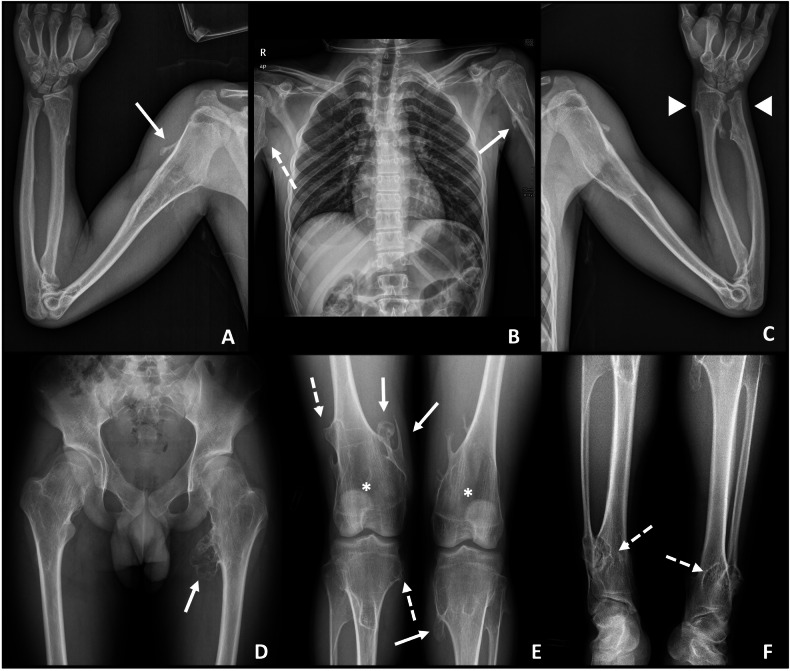
A 14-year-old male diagnosed with HME. Skeletal survey with conventional radiography (**A**–**F**). Multiple osteochondromas are present (more than 50), located in the lower limbs (**D**–**F**), upper limbs (**A**–**C**), and pelvis (**D**). Some of them are pedunculated (continuous arrows) and others are sessile (dashed arrows). Metaphyseal widening is seen in the femora (Erlenmeyer flask deformity) (*) and tibiae. In the left distal radioulnar joint, both radial and ulnar osteochondromas are observed (arrowhead), with ulnar shortening (pseudo-Madelung deformity), all of it consistent with a deformity type IV-A of the Masada-Jo classification. In the legs, osteochondromas are present in both the proximal and distal tibiofibular joints. This patient belongs in the group A of the Ahn classification, predisposing to deformity in this location

**Fig. 2:**
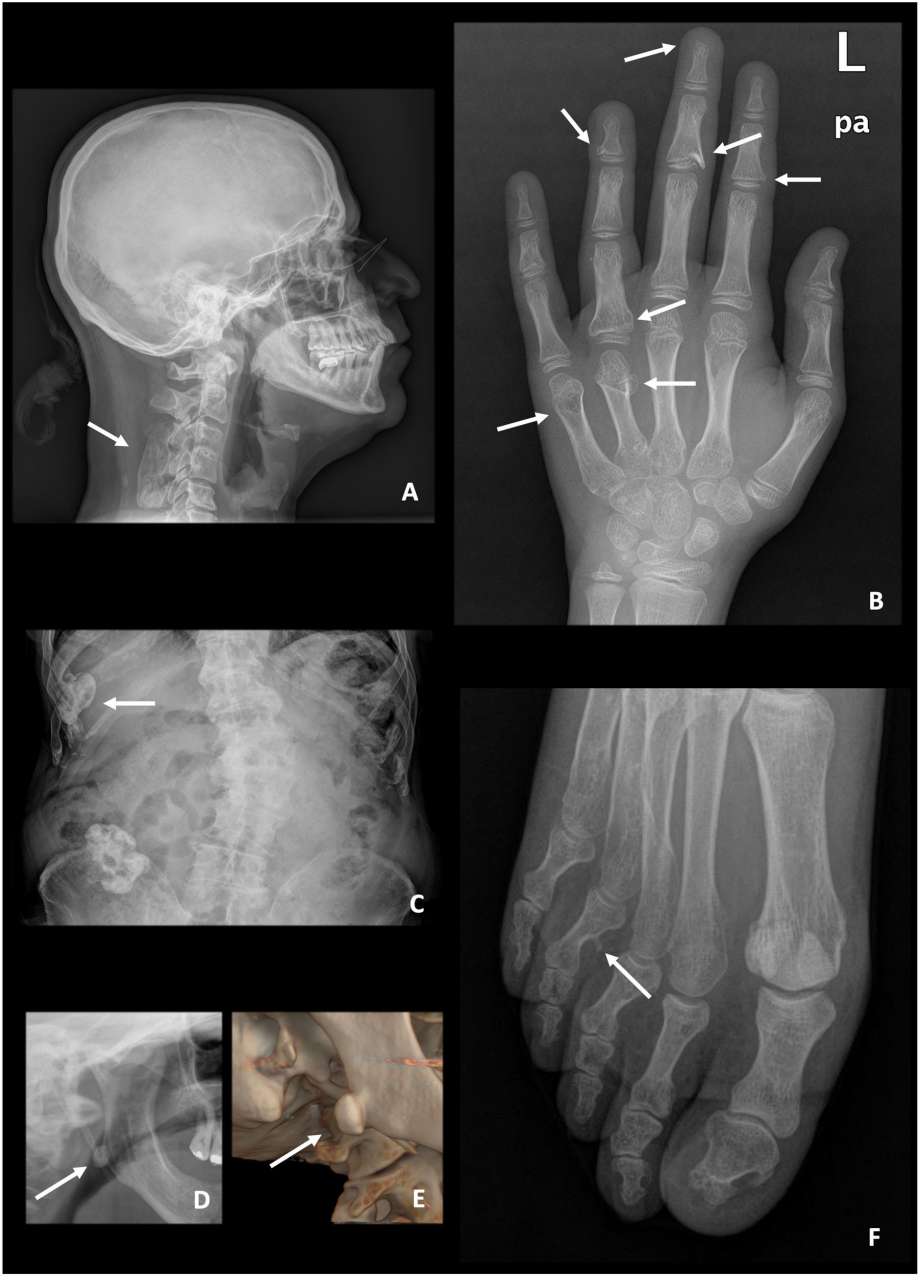
Less common locations of osteochondromas (arrows) in different patients with HME. **A** Cervical spine of a 54-year-old male. **B** Hand of an 8-year-old girl. **C** Ribs of an 82-year-old male. **D**, **E** Mandible of a 58-year-old female, with a 3D reformat shown. **F** Foot of a 54-year-old male

With respect to location, osteochondromas usually occur in the long bones of the lower extremities, most often centred around the pelvic and knee joints. Lesions of the upper extremities are less frequent. Less commonly, injuries can arise from the scapula, pelvis, ribs, sternum, spine, and bones of the hands and feet [1, 4, 16–18] (Figs. 1 and 2) (Table 1).

**Table 1:** Distribution and frequencies of osteochondromas in HME (Murphey 2000 [1] and Guo 2014 [4])

Location	Prevalence, (%)
Scapula and ribs	40
Spine	< 5
Pelvis	5–15
Humerus	50–98
Elbow	35–40
Wrist	30–60
Hands	20–30
Hips	30–90
Knees	70–98
Ankles	25–54
Feet	10–25

**Fig. 3:**
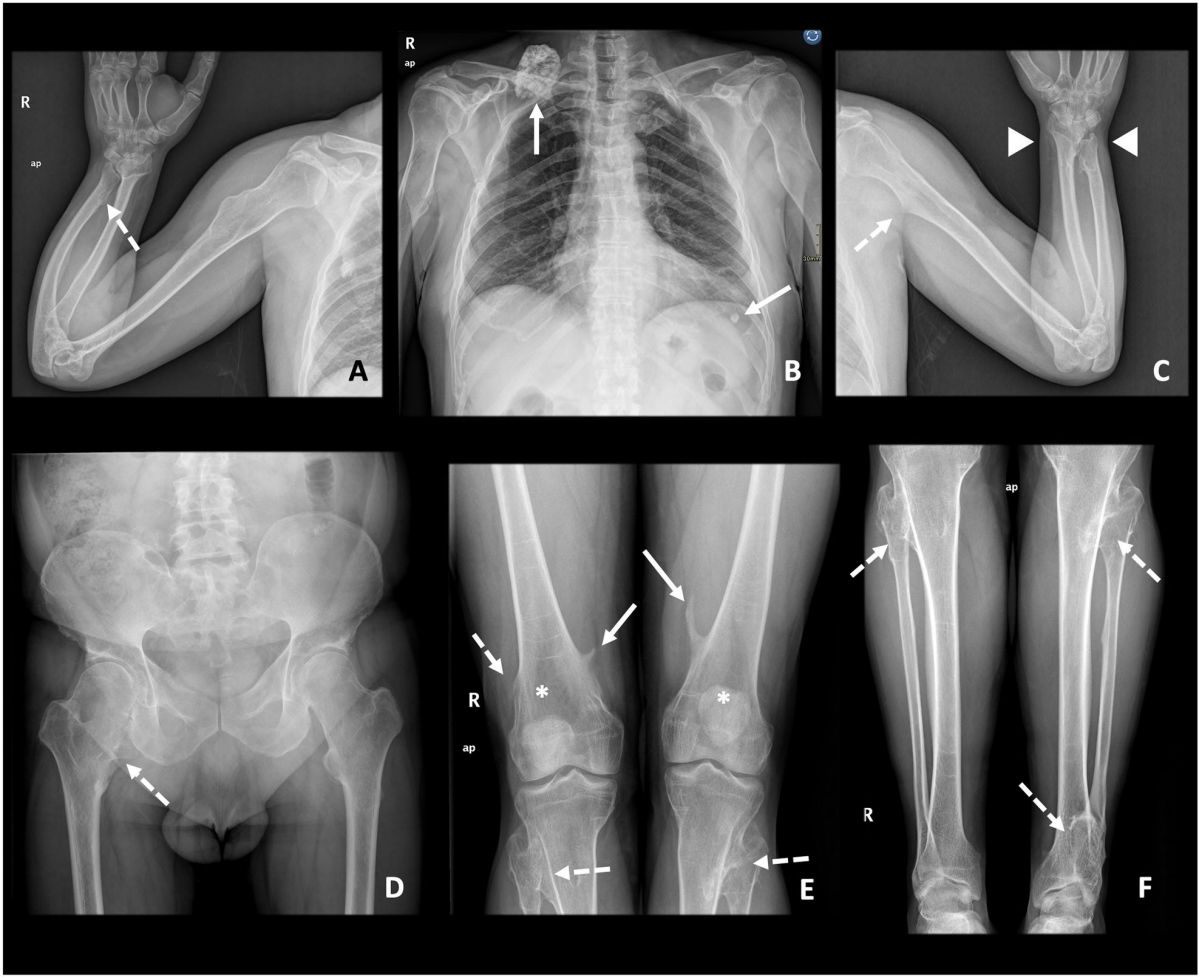
A 54-year-old male diagnosed with HME. Skeletal survey with conventional radiography (**A**–**F**). Multiple osteochondromas are present, located in the lower limbs (**D**–**F**), upper limbs (**A**–**C**), scapula and ribs (**B**), and pelvis (**D**). Some of them are pedunculated (continuous arrows) and others are sessile (dashed arrows). Metaphyseal widening is seen in the femora (Erlenmeyer flask deformity) (*) and tibiae. In the left distal radioulnar joint, both radial and ulnar osteochondromas are observed (arrowhead), with ulnar shortening (pseudo-Madelung deformity), all of it consistent with a deformity type IV-A of the Masada-Jo classification. In the right radioulnar joint there is an ulnar osteochondroma with concomitant ulnar shortening, consistent with a deformity type I of the Masada-Jo classification. In the legs, osteochondromas are present in both the proximal and distal tibiofibular joints. This patient belongs in the group A of the Ahn classification, predisposing to deformity in this location

Patients with HME tend to have a height below the 5th percentile, predominantly due to the shortening of the limbs rather than the shortening of the axial skeleton [19] (Fig. 3). Multiple deformities associated with HME have been described. Deformities of the forearms and ankles are very characteristic and various authors have proposed classification systems to guide follow-up and treatment, such as the Masada-Jo classification for deformities of the forearm [20, 21] and the Ahn classification for deformities of the leg and ankle [22]. All of them will be further described later. The severity of the clinical presentation, including associated malformations, depends on the number, size and location of the osteochondromas [1, 15].

## Pathogenesis and histopathology

HME is an autosomal dominant disease with incomplete penetrance in females and multiple associated genetic mutations, usually in the EXT (exostosin) family [15, 23–25].

The EXT family are tumour suppressor genes. They encode glycosyltransferases, which are vital for the synthesis of heparin sulphate (HS), a polysaccharide material that binds to core proteins to produce heparin sulphate proteoglycans (HSPGs) [26]. HSPGs are involved in the regulation of bone and cartilage formation [27].

Heterozygous mutations of the EXT-1 or EXT-2 genes lead to an HS deficiency of approximately 50%. However, this is insufficient to lead to the formation of osteochondromas and a second genetic impact, such as loss of heterozygosity or aneuploidy, is required to further reduce HS levels, causing tumourigenic cells to be produced [28].

Mutations are not present in IDH-1 or IDH-2 genes (isocitrate dehydrogenase), which are characteristic in central cartilaginous tumours [5, 29].

Mutations in the EXT-1 gene are responsible for half of HME cases, while the EXT-2 gene is implicated in one-third of cases [2], with mutations in these genes having been identified in up to 94% of cases overall [30]. Variability between different ethnicities has been described [4]. Although it has not been frequently evaluated, small studies have found EXT-1 and EXT-2 mutations in patients with solitary osteochondromas, suggesting a possible similar genetic link [1, 27]. An EXT-3 gene has been sequenced and it plays a vital role in HS synthesis, but no such mutations have been detected in patients with HME [31].

Francannet et al described that patients with mutations in EXT-1 are more severely affected, as they develop more osteochondromas, suffer more severe symptoms and are more likely to develop sarcomatous degeneration [32].

Variations in clinical presentation are multifactorial and may depend on the genetic mutation, variation in penetrance, as well as gender, and is an area of ongoing research.

Osteochondromas develop through endochondral ossification. They consist of three layers: an outer perichondrium, and a hyaline cartilaginous cap with underlying endochondral ossification which is continuous with the underlying medullary bone. This continuity of marrow assists in distinguishing it from other superficial bone tumours, such as bizarre paraosteal osteochondromatous proliferation [5].

Embryologically, these lesions result from the separation of a fragment of epiphyseal growth plate cartilage, which subsequently herniates through the periosteal bone cuff that normally surrounds the growth plate [1].

The cartilaginous cap of an osteochondroma continues to grow throughout puberty until epiphyseal closure. Consequently, any peripheral osteocartilaginous tumour emerging in a skeletally mature patient with a cartilaginous cap of 15–20 mm or more should raise suspicion of malignant transformation [1, 5, 33].

Failure of normal tubulation, especially in the metaphysis, can sometimes be the first manifestation of HME on imaging, and is an easily identifiable finding on conventional radiographs. This frequently occurs in the hip and knee, resulting in a widening of the metadiaphyseal junctions that may appear as an Erlenmeyer flask deformity [1].

## Imaging features

In addition to a detailed anamnesis and a thorough clinical examination, imaging techniques are required for the diagnosis of osteochondroma [6]. The key radiological features of osteochondroma are cortical and medullary continuity between the lesion and the underlying bone, and a cartilaginous cap [34].

Osteochondroma is usually diagnosed only by radiographs, especially if located in the metaphysis of long bones [1]. However, complex lesions or lesions located in anatomical regions that are difficult to assess, such as the shoulder girdle, pelvis or spine, may be better characterised by CT or magnetic resonance imaging (MRI) [10].

### Conventional radiography

The skeletal survey for HME assessment includes radiographs of the femora, tibiae, humeri and forearms, as well as the thorax, spine, and pelvis. It is usually performed on a yearly basis in patients with a diagnosis of HME. Other candidates for a skeletal survey include patients with two or more osteochondromas, patients with osteochondroma and osseus deformities that suggest a diagnosis of HME and patients with genetic mutations associated with the diagnosis of HME. Areas of apparent deformity can be further studied with targeted radiographs [5].

On conventional radiography, osteochondromas appear as sessile (broad-based) or pedunculated (narrow-based) metaphyseal protrusions projecting out of the epiphysis. Cortical and medullary continuity is difficult to assess with this technique but can occasionally be seen in lesions arising from long bones and even more so in pedunculated lesions. A possible differential diagnosis to consider on plain radiography is parosteal osteosarcoma, which appears as an exophytic lobular mass, often highly ossified, with irregular margins and cortical continuity with the underlying bone, but lacks the medullary continuity that is pathognomonic of an osteochondroma [34, 35].

The overlying cartilaginous component can sometimes be seen on radiography, and, if thickened, chondroid matrix mineralisation may be visible in a classic “ring-and-arc” pattern, but the thickness cannot be adequately measured [1, 17].

The limitations of radiography are mainly due to the inability to assess the surrounding soft tissues and secondary complications. Some anatomical regions, where obtaining two orthogonal projections may be difficult can require further imaging to be evaluated too [1, 5].

### Computed tomography (CT)

**Fig. 4:**
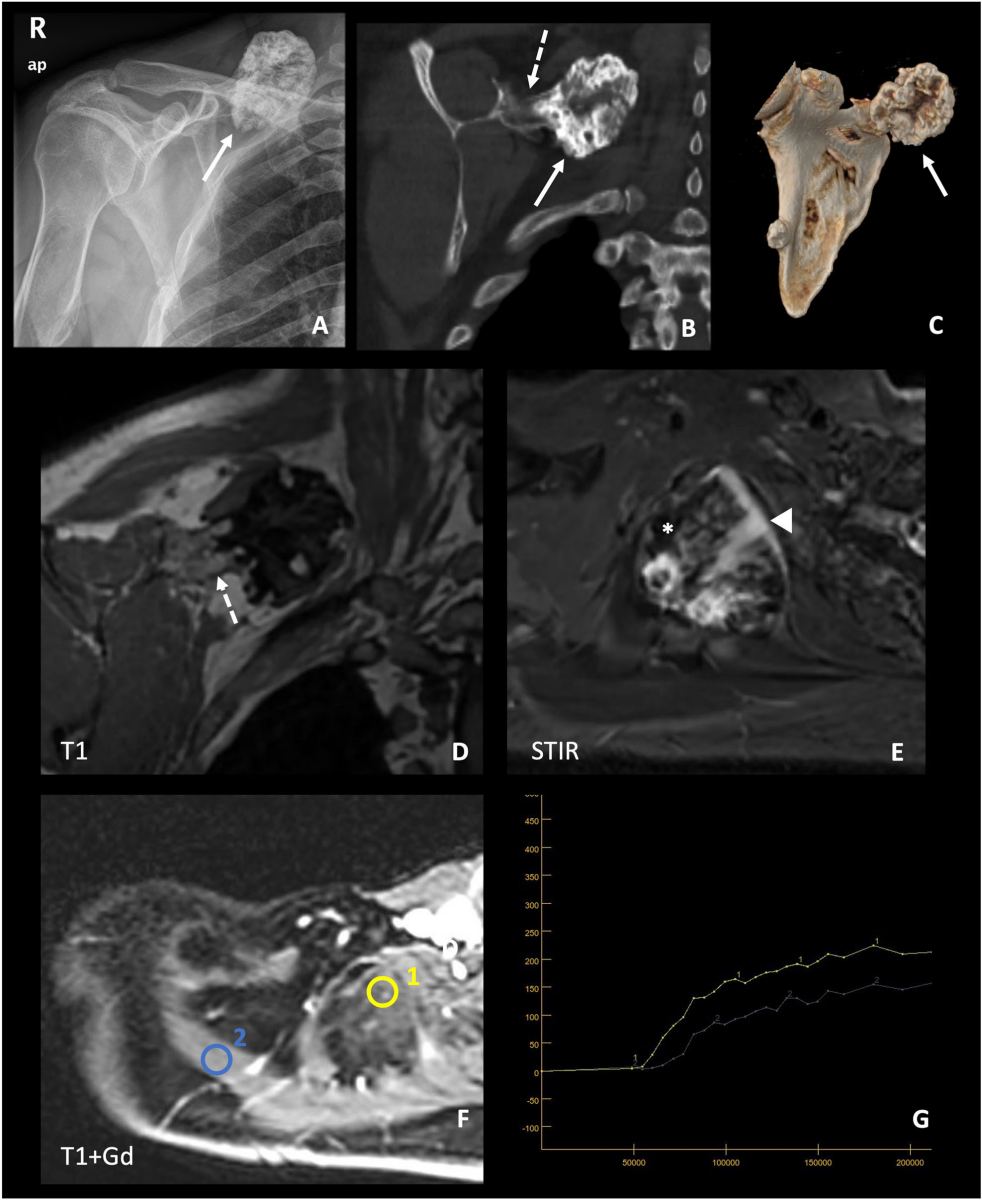
Detailed study of an osteochondroma in the right scapula of the 54-year-old male from Fig. 3. Targeted plain radiograph (**A**) and CT images: coronal reconstruction (**B**) and 3D volumetric reconstruction (**C**). MRI: T1W coronal plane (**D**), STIR axial plane (**E**), post-contrast dynamic study, with reference image (**F**), and curves of enhancement (**G**). Some characteristic features of the osteochondroma can be seen, such as the partially calcified cartilage cap with a ring-and-arc pattern (solid arrows). The cortical and medullary continuity with the underlying bone (dashed arrows) is key for making a precise diagnosis of osteochondroma. The cartilaginous cap shows high SI in STIR (arrowhead), with foci of calcification (low SI in STIR) (*). The cap thickness was 10 mm, not suspicious of malignancy, but surveillance was recommended. In the dynamic post-contrast study, the cartilaginous cap (ROI no. 1) presents an enhancement curve type II (progressive enhancement), which is similar to that of the ipsilateral supraspinatus muscle (ROI no. 2) and suggests a benign lesion

The three-dimensional nature of CT allows the characterisation of lesions in more complex locations, such as the shoulder, scapula, spine and pelvis, while allowing some assessment of surrounding soft tissue structures. In addition, it is superior to radiography in assessing the morphology of osteochondromas, demonstrating cortical and medullary continuity with the underlying bone more definitively [1, 6] (Fig. 4).

The appearance of the cartilage cover is variable on CT, and accurate assessment of thickness is an area of controversy, although superior to plain radiographs [36]. The accuracy of cartilage thickness assessment on CT is highly dependent on the degree of calcification of hyaline cartilage, as non-mineralised cartilage can be difficult to distinguish from other adjacent structures on CT [1, 10].

CT with 3D reconstruction provides an excellent anatomical representation of various limb deformities, which is useful in the context of pre-surgical planning [5].

In an emergency setting, the availability of CT is ideal for the assessment of complications, such as spinal cord compression, pneumothorax, pseudoaneurysm and fracture [37].

The main drawback of CT is its radiation dose and it should be avoided in screening and follow-up, especially in young patients, due to the overall radiation burden [10, 38].

### Ultrasound

**Fig. 5:**
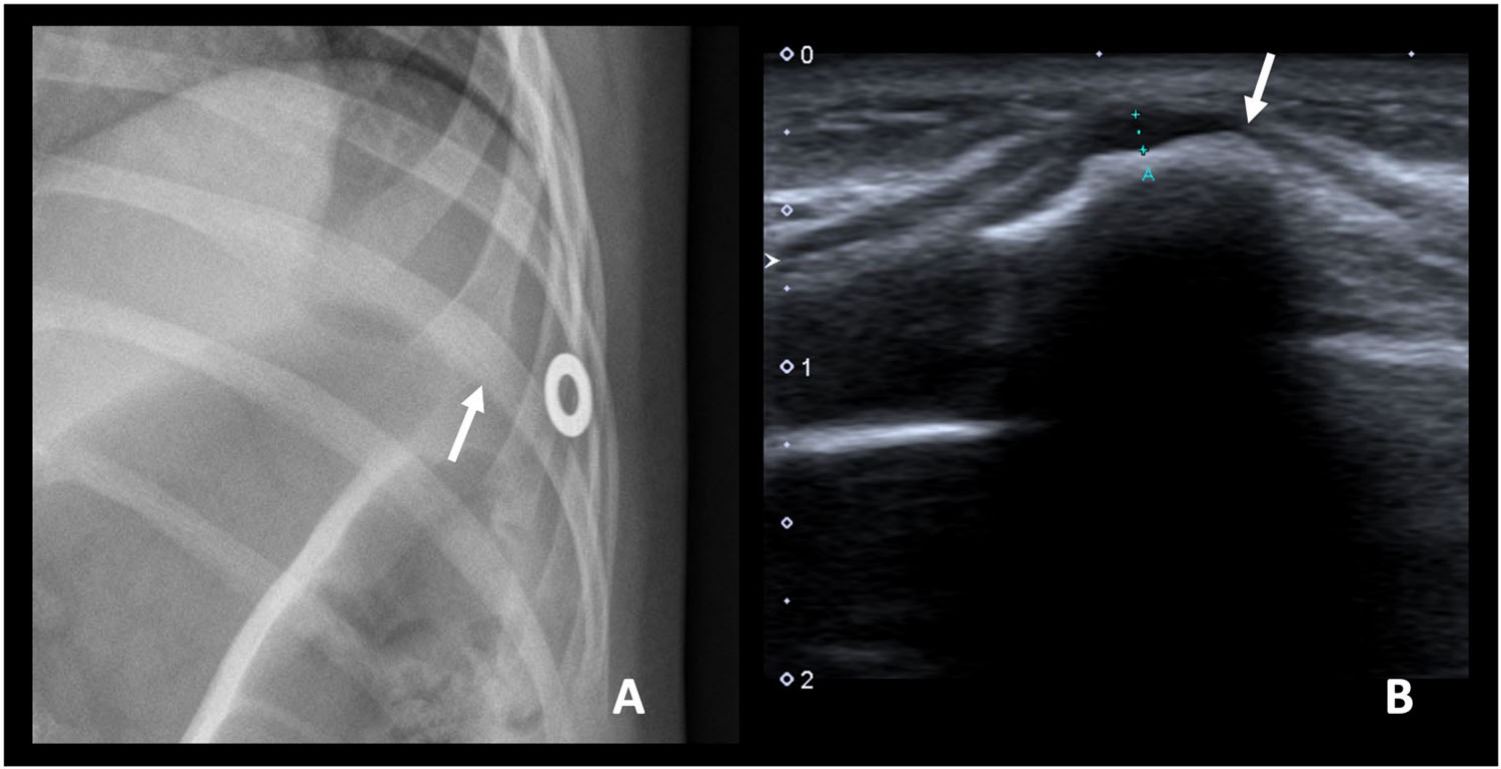
An 8-year-old girl diagnosed with HME that consulted for a palpable mass in the anterior chest wall. Conventional radiography of the ribs with a metallic marker (**A**) and ultrasound (**B**) revealed an osteochondroma in the anterior arch of a left rib (arrows). The cartilage cap measured 2 mm, within the normal range

Ultrasound can provide precise evaluation of the cartilaginous cap of superficial lesions, presenting as a hypoechoic region overlying the bony protrusion, with areas of cartilaginous mineralisation displaying posterior acoustic shadowing. Its accuracy is comparable to MRI, with an average error rate of less than 2 mm in cartilage lesions measuring up to 20 mm [39]. It is especially useful in children, where the possibility of performing an MRI may be more limited. However, ultrasound has limitations, including its reliance on the operator and its inability to assess deeper lesions and the bony component of the lesions [1] (Fig. 5).

### MRI

MRI facilitates accurate evaluation of the cartilaginous cap and provides an excellent assessment of the surrounding structures. The non-mineralised regions of the cartilage cap exhibit a high water content and should demonstrate signal intensity (SI) characteristics consistent with normal hyaline cartilage across all pulse sequences: intermediate to low SI on T1-weighted images (T1WI) and high SI on T2-weighted images (T2WI) and short tau inversion recovery (STIR) sequences, and fat-suppressed fast spin echo (FSE) T2WI sequences. In adults, the cap thickness should typically be less than 15–20 mm, with thicker caps raising suspicion of malignancy. In benign lesions, gadolinium studies should not reveal cap enhancement, although septal and peripheral enhancement falls within the normal range [40] (Fig. 4).

The role of diffusion-weighted imaging (DWI) and whole-body MRI in HME will be further discussed in the malignant transformation section below.

Due to the absence of ionising radiation and superior soft tissue contrast, MRI is the preferred imaging modality for examining complex lesions and assessing secondary complications in HME. MRI outperforms radiography and CT in illustrating the cortical and medullary continuity of osteochondromas with the bone of origin [6, 41].

The main disadvantages of MRI include longer image acquisition times, contraindications for patients with claustrophobia or metal implants, and the potential need for sedation in children.

### Nuclear medicine

Scintigraphy evaluates the metabolic activity of a tumour and detects new bone lesions by imaging the entire body in a single examination [42]. A normal isotopic bone scan eliminates the possibility of malignant transformation of an osteochondroma, while a positive result cannot distinguish the endochondral ossification seen in benign osteochondroma from the hyperaemia and osteoblastic reaction characteristic of chondrosarcoma [43].

[^18^F]FDG PET imaging may have a role in the differential diagnosis and classification of chondrosarcomas. However, during childhood and adolescence, abnormal uptake of fluorodeoxyglucose may occur due to the growth of exostoses. The limitations of FDG-PET include the use of ionising radiation, restricted availability, high cost and the fact that it is a less explored field [44, 45].

## Complications

Complications associated with osteochondromas are frequent and include compression of adjacent structures, fractures, bone deformities, bursa formation, with or without bursitis, and malignant transformation [11, 12, 46]. Malignant transformation is more frequent in HME than in solitary osteochondromas [8].

### Non-malignant complications

#### Osseous deformities (Table 2)

Deformities resulting from osteochondromas in HME can manifest throughout the skeleton, although they are most frequently observed in the forearm and lower extremities. Research indicates that deformities in these areas are directly related to the overall extent of the disease. Carroll et al described that the severity of deformities correlates with the number of osteochondromas [15].

**Table 2:** Common osseus deformities in HME (Murphey 2000 [1], Ellatif 2021 [5], and Ahn 2019 [22])

Location and deformity	Prevalence (%)
Forearm and wrist	40–74
Hip: *coxa valga* and others	25
Knee: *genu valgus*	8–33
Ankle: *valgus* deformity and others	45–73
Limb length discrepancy	10–50
Short stature	40

##### Adjacent bones erosion

**Fig. 6:**
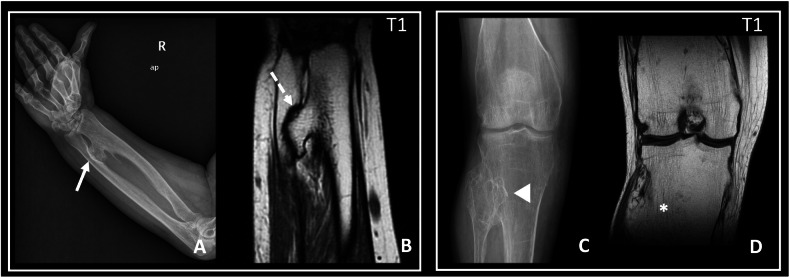
Neighbouring bone erosion and synostosis in HME: illustrative cases. **A**, **B** Eighty-two-year-old woman diagnosed with HME. **A** Conventional radiograph of the right forearm shows a radial osteochondroma which contacts with the ulna (solid arrow). **B** Coronal T1W image showing the radial osteochondroma causing erosion and cortical scalloping in the ulna (dashed arrow). Note: the radial osteochondroma without associated ulnar osteochondroma is consistent with a type III Masada-Jo deformity. **C**, **D** Young male diagnosed with HME. **C** Conventional radiograph of the right knee showing proximal radial and fibular kissing osteochondromas (arrowhead). **D** Coronal T1W image showing medullar continuity between both lesions, consistent with synostosis

Pressure erosions on adjacent bones related to compression caused by osteochondroma are more prevalent in larger lesions, especially in the forearm or lower leg. This compression often leads to scalloping of the compressed cortex in the adjacent bone [1] (Fig. 6).

##### Synostosis

Synostosis between adjacent bones is a well-recognised complication of osteochondromas, attributed to the bony fusion of two contiguous lesions, known as “kissing osteochondromas”. Individuals with HME may exhibit multiple foci of synostosis, which can either be asymptomatic or present symptoms such as nerve or tendon entrapment. Synostoses are seldom observed in the forearm due to the wide range of motion between the radius and ulna, making them more frequent in the lower extremities. While proximal tibiofibular synostosis is the most commonly described in HME, distal lesions are also documented [5, 47] (Fig. 6).

##### Forearm deformities

Up to 40–74% of patients with HME have forearm deformities due to osteochondromas [48–50]. They are often associated with ulnar shortening and radial bowing with dislocation of the radial head, which may result in loss of pronation. Dislocation of the distal radioulnar joint and ulnar translocation of the carpal bones may also be present. This spectrum of forearm deformities has been associated with sessile exostoses rather than pedunculated lesions and it has been suggested that their severity is proportional to the size of the lesion [15].

**Fig. 7:**
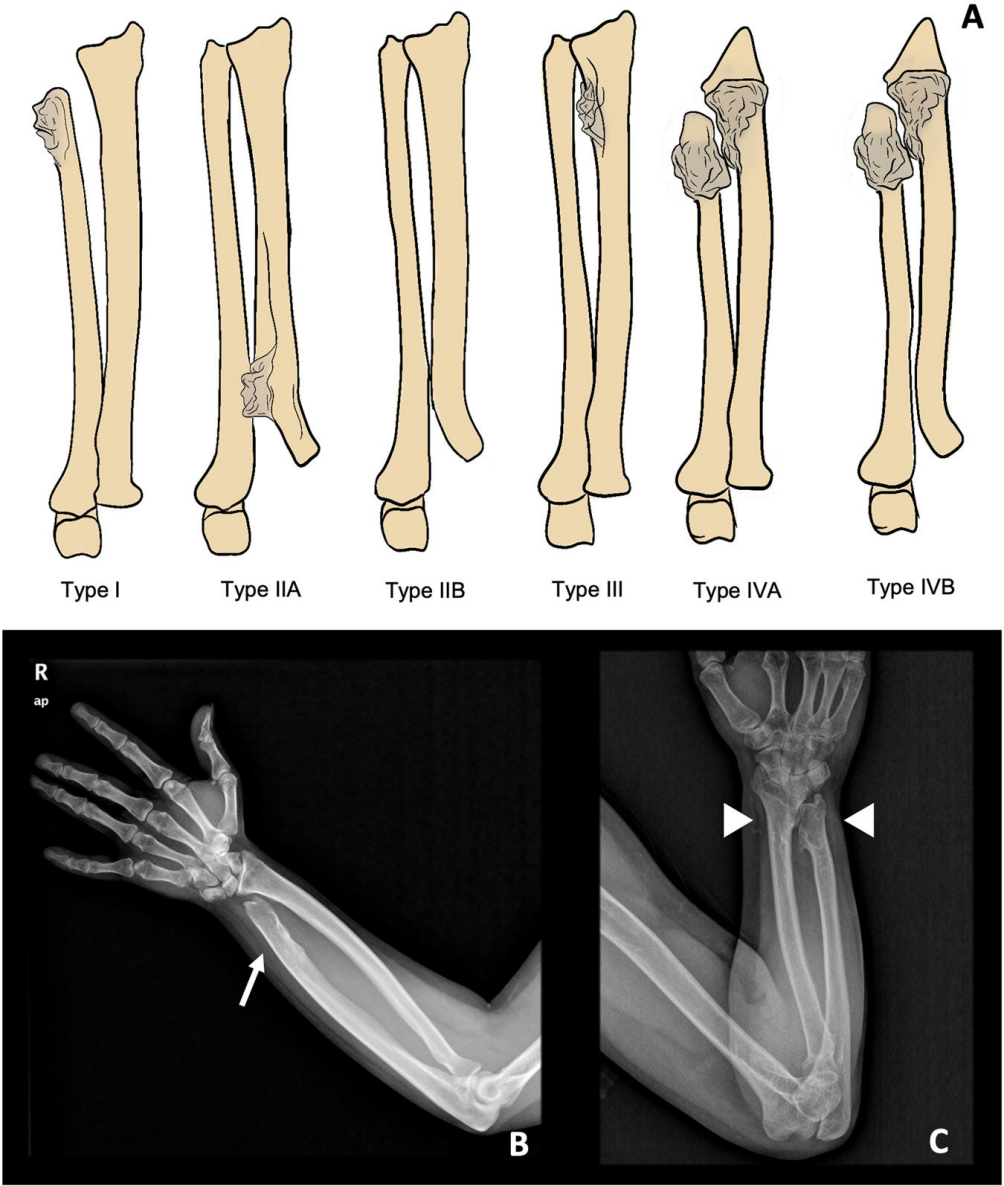
**A** Masada-Jo classification for forearm deformities in patients with HME. Illustrative cases: **B** Ulnar osteochondroma (arrow) with ulnar shortening and bowing, consistent with a type I deformity. **C** Ulnar and radial distal osteochondromas, without radial head dislocation, consistent with a type IV-A deformity

Masada et al [20] suggested in 1989 a classification of distal forearm deformity, in which type I represents an isolated distal ulnar lesion with associated ulnar shortening, type II is the presence of shortening in addition to radial head dislocation, and type III is radial exostosis with radial shortening. Subsequently, Jo et al [21], recently proposed a modified classification, which adds a group of deformities previously unclassifiable under Masada, classifying them as type IV. These are injuries arising from both the distal ulna and radius, with or without radial head dislocation. In clinical practice, Masada and Jo’s classifications are the most widespread for assessing forearm deformities. They guide orthopaedic surgeons in assessing the need for surgical treatment of children with HME, as well as in deciding the type of surgery and the timing for it, to allow for proper development and growth of the patient. However, recent studies such as that of Canizares et al [51] suggest that they may be unreliable classifications, since interobserver correlation was low for both. The need for a new classification for forearm deformities in HME is currently an open field of research [52] (Table 3 and Fig. 7).

**Table 3:** Masada-Jo classification for forearm deformities in patients with HME [20, 21]

Type	Description
Type I	Osteochondroma(s) in the distal ulna, with associated ulnar shortening (Figs. 3A and 7B)
Type II-A	Radial head dislocation with osteochondroma(s) in proximal radius
Type II-B	Radial head dislocation without osteochondroma(s) in proximal radius
Type III	Osteochondroma(s) in distal radius (Fig. 6A)
Type IV-A	Osteochondroma(s) in both distal ulna and radius without radial head dislocation (Figs. 1C, 3C, and 7C)
Type IV-B	Osteochondroma(s) in both distal ulna and radius with radial head dislocation

##### Limb length discrepancies

Short stature and limb length discrepancy are prevalent in HME and may arise from femoral or tibial shortening due to exostoses occurring during childhood and early puberty. Femoral shortening is twice as frequent as tibial shortening, with reported discrepancies of over 2 cm in 10–50% of patients. Surgical intervention typically involves epiphysiodesis performed at the appropriate time to halt the growth of the physis [2, 19, 50].

##### Hip deformities

Femoral deformities are reported in 30–90% of patients with HME, with *coxa valga* noted in up to 25% [2, 53]. Surgical correction for *coxa valga* typically involves early varus osteotomy. Acetabular dysplasia can occur due to exostoses within the acetabulum or in close proximity to the medial aspect of the femoral neck, leading to lateralisation of the femoral head [19, 53, 54]. Hip joint subluxation, primarily seen in young children, has an incidence of 5–19% [55].

Lesions affecting the lesser trochanter have been associated with valgus deformity and femoral anteversion. Ischiofemoral impingement, a common presentation in patients with HME experiencing hip pain, typically occurs in young patients due to an osteochondroma originating from the lesser trochanter of the femur or the ischial tuberosity, leading to narrowing of the ischiofemoral space. MRI can identify the lesion and reveal the presence of oedema or fatty atrophy of the ipsilateral quadratus femoris muscle [56, 57].

##### Knee and ankle deformities

Valgus deformity of the knee affects 8–33% of cases of HME and is typically attributed to proximal tibial injuries, with less common occurrences involving distal femoral and proximal fibular injuries [5].

Ankle exostoses can lead to deformity, restricted range of motion, and pain. Valgus deformity of the ankle joint is observed in approximately 50% of patients with HME, primarily due to fibular shortening, which can also result in medial subluxation of the talus [50].

**Fig. 8:**
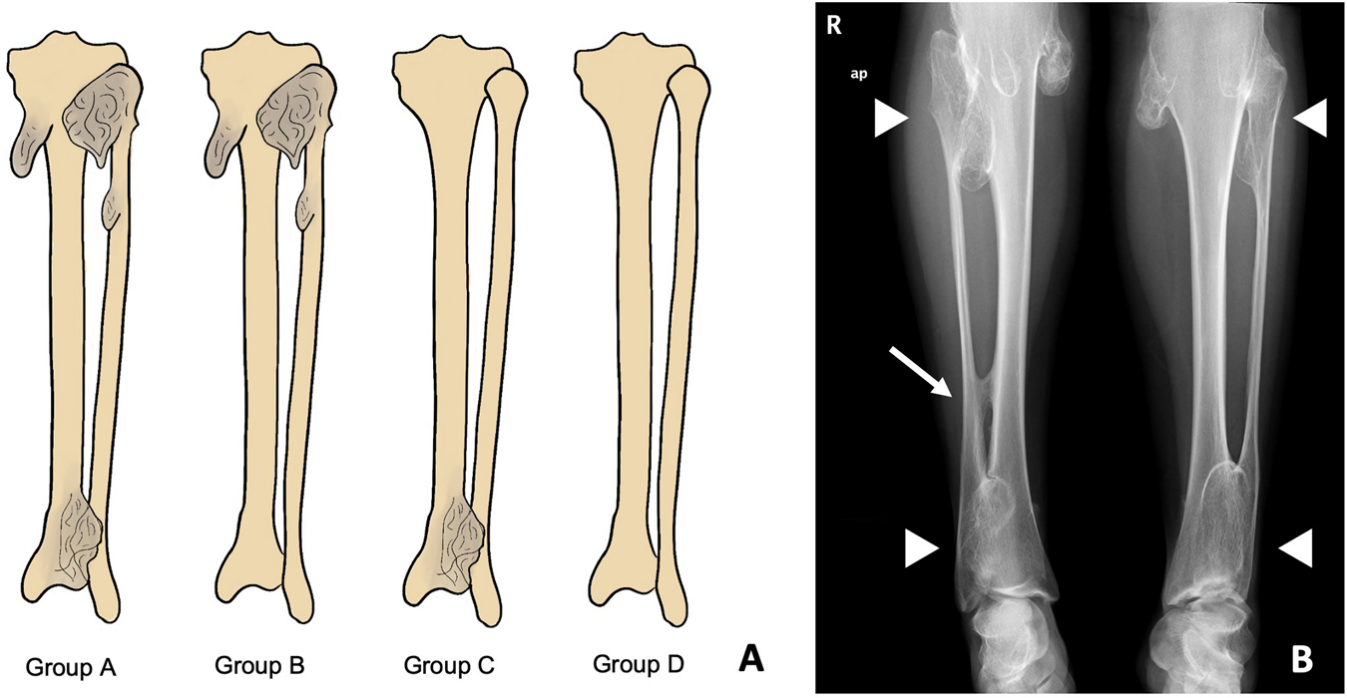
**A** Ahn classification for tibiofibular coronal malalignment risk stratification in patients with HME. **B** Illustrative case. Both legs of this young female belong in group A, since both proximal and distal tibiofibular are affected (arrowheads). The arrow marks a tibiofibular synostosis in the right leg

A classification proposed by Ahn et al [22] aims to assess the degree of coronal malalignment of ankles and knees. This predicts the risk of future development of valgus deformities in these patients and helps orthopaedic surgeons plan possible treatments according to that risk. The classification consists of four groups: group A comprises patients with lesions affecting both proximal and distal tibiofibular joints, group B includes those with only proximal lesions, group C involves distal tibiofibular joint involvement, and group D entails no joint involvement (Table 4 and Fig. 8).

**Table 4:** Ahn classification for tibiofibular coronal malalignment risk stratification in patients with HME [22]

Group	Description
Group A	Both proximal and distal tibiofibular joints were affected (Figs. 1, 3, and 8B)
Group B	Only the proximal tibiofibular joint is affected by osteochondromas
Group C	Only the distal tibiofibular joint affected by osteochondromas
Group D	Both proximal and distal tibiofibular joints free of osteochondromas

Patients with osteochondromas and associated ankle deformities face an increased risk of early secondary osteoarthritis, with reported rates of up to 19% at 42 years of age according to Noonan et al [48]. Therefore, surgical intervention for symptomatic lesions at this site is recommended for both skeletally mature and skeletally immature patients [6].

#### Neural impingement and spinal abnormalities

Up to 23% of patients with HME experience symptoms of peripheral nerve root compression [25]. Proximal fibular injuries leading to compression of the common peroneal nerve and subsequent neuropathy and foot drop are well-documented complications. Additionally, radial nerve palsy resulting from humeral compression injury has been reported. Since nerves are often too small to be reliably identified by imaging, secondary atrophy and fatty infiltration of muscle groups, detectable on MRI, are frequently used as indirect signs [5].

Central osteochondromas originating from the skull, vertebral bodies, or ribs may manifest a range of symptoms, from cranial nerve neuropathy to cauda equina syndrome. In vertebral bodies, they primarily affect the posterior elements, although anterior-directed spinal cord compression is rare. The cervical spine is most frequently affected. Some authors have also documented scoliosis caused by osteochondromas [58–60].

#### Vascular abnormalities

Osteochondromas close to vascular structures can lead to clinically significant compression and have been reported in up to 11.3% of cases [25]. This compression may manifest as vascular compression, arterial or venous thrombosis, or pseudoaneurysm formation, resulting in clinical claudication, acute ischaemia, or thrombophlebitis. Additionally, recent reports have indicated an association with venous malformations [61].

These complications are more commonly observed in the lower limbs, particularly around the knee, where they involve the popliteal vessels. Pseudoaneurysm formation due to osteochondromas can affect various arteries including the popliteal, posterior tibial, femoral, and brachial arteries. Thoracic outlet syndrome resulting from bilateral exostoses at the base of the neck has also been documented [5, 14].

#### Bursa formation

Bursa formation between an osteochondroma and the surrounding soft tissues occurs in approximately 1.5% of all osteochondromas due to overlying motion and friction [1]. It is most frequent in lesions involving the scapula and bones around the shoulder joint. The bursa is lined with synovium and may become infected, inflamed, or bleed, resulting in a painful mass that increases in size. If chondrometaplasia develops, it may lead to secondary chondromatosis, presenting as either a painless or painful, slow-growing mass [5, 62].

**Fig. 9:**
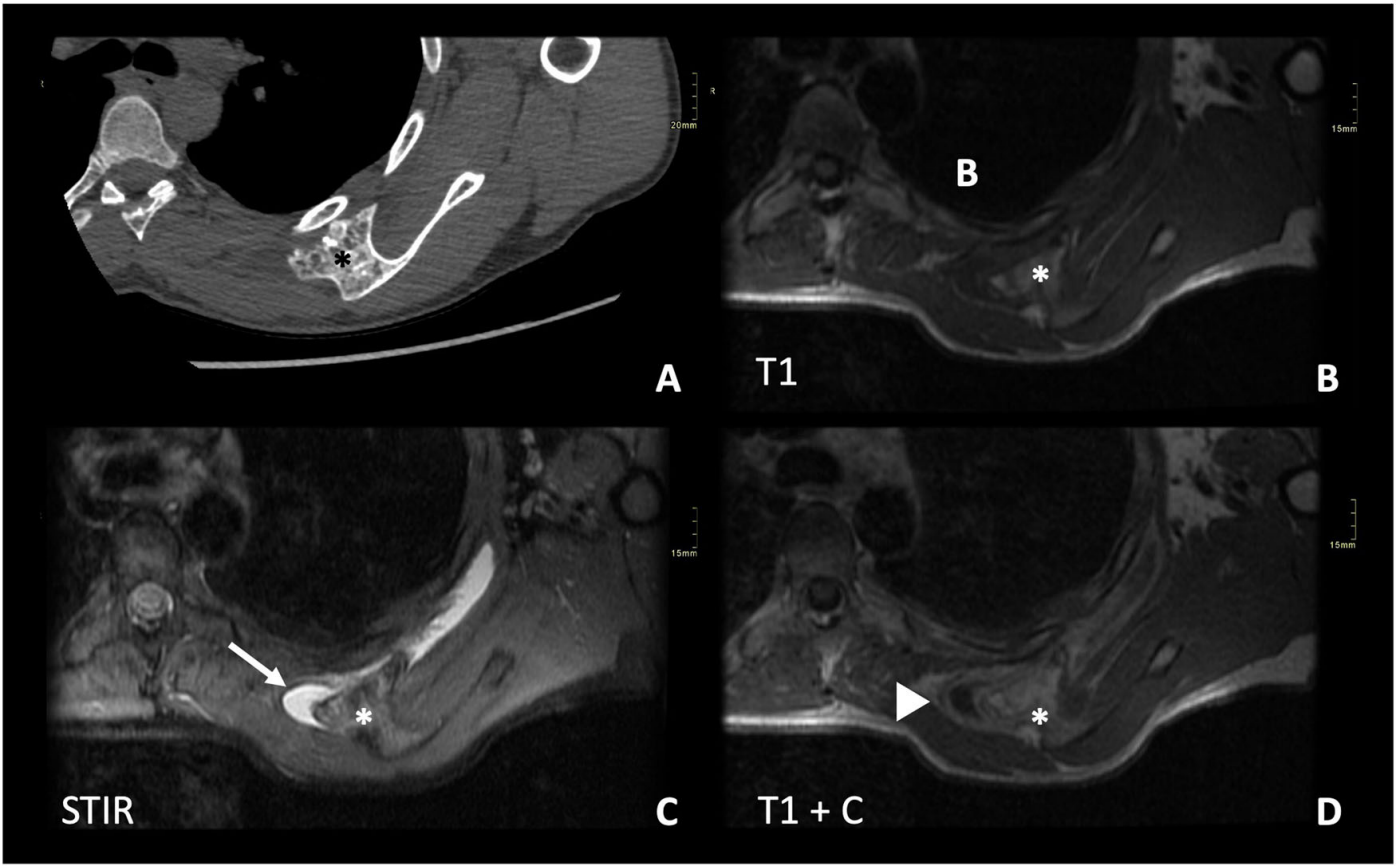
A 28-year-old male diagnosed with HME presents with pain in his left shoulder. Axial CT scan (**A**) and MRI with axial T1W (**B**), STIR (**C**), and T1W post-contrast T1 (**D**) sequences. There is an osteochondroma (*) in the scapula, showing key features such as cortical and medullary continuity with the underlying bone. There is a soft tissue mass surrounding it, with low SI in T1 and high SI in STIR, consistent with a bursa (arrow). The post-contrast sequence shows enhancement of the wall of the bursa (arrowhead), which suggests inflammation

Distinguishing between a bursa and malignant transformation is crucial, especially in the context of a rapidly growing painful mass. Radiographs cannot reliably differentiate between them, as both may appear as soft tissue lesions with possible chondroid calcification. US of superficial lesions typically reveals an anechoic bursa with associated posterior acoustic enhancement, independent of the underlying osteochondroma. Cross-sectional CT or MR imaging will demonstrate a fluid-filled lesion, different from the cartilage cap of the osteochondroma, with or without enhancement in exams with contrast depending on whether the bursa is inflamed or not [5, 12] (Fig. 9).

#### Fracture

**Fig. 10:**
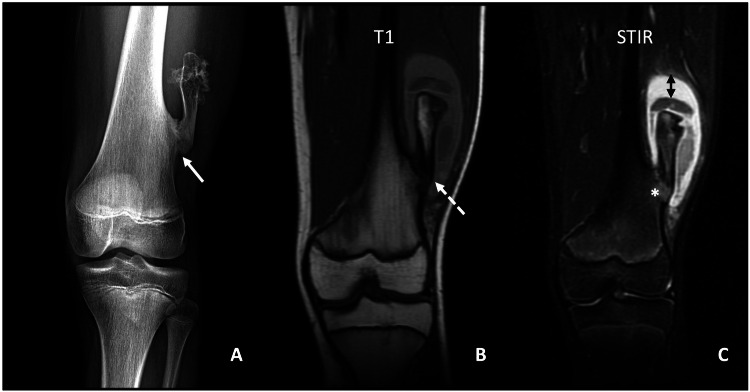
14-year-old male with a history of osteochondroma on the lateral aspect of the left distal femur, who consulted for pain after trauma. Conventional radiograph (**A**), coronal T1W MRI (**B**), and coronal STIR (**C**), showing the left distal femoral osteochondroma, with a radiolucent trace at the base (continuous arrow), compatible with fracture, which is also identified in the T1W sequence (dashed arrow). In the STIR sequence, there is an area of bone marrow oedema (*). Note the thick cartilage cap (< 2 cm thick) (double arrow). Resection of the osteochondroma was performed, with no signs of malignancy in the anatomopathological study

Localised trauma to the site of an osteochondroma can result in a fracture through the lesion, typically occurring through the base of the peduncle of a pedunculated exostosis. This phenomenon is most frequently observed in the knee. Callus formation occurs during lesion healing. If a fracture occurs without a history of trauma, further testing is indicated to rule out malignant transformation of the osteochondroma [37, 63] (Fig. 10).

#### Other

**Fig. 11:**
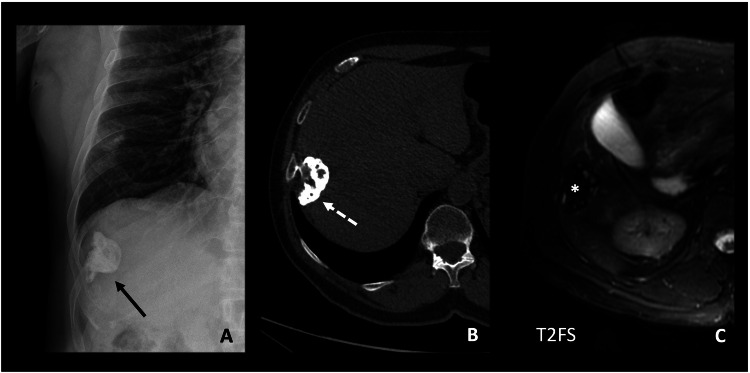
A 70-year-old man with a history of HME underwent abdominal ultrasound for elevated transaminases and a liver mass with very poorly defined contours was identified (not shown). Plain radiography (**A**), non-contrast CT (**B**), and MRI (**C** T2 with fat suppression) were performed. An osteochondroma dependent on the right 9th rib is identified, with calcification of the cartilaginous cap (continuous and dashed arrows on CT). The CT scan shows cortical and medullary continuity with the rib. The MRI shows hyperintense foci in T2, corresponding to the non-mineralised cartilaginous cap, and hypointense foci in T2, which correspond with the calcified cap visible on the CT (*). Since the patient was asymptomatic, a conservative approach was decided

Gastrointestinal and genitourinary tract disorders secondary to osteochondromas have been documented, leading to symptoms such as dysphagia, haematuria, bladder outflow obstruction, or compression of other abdominal viscera (Fig. 11). Pelvic osteochondromas in pregnant women may also result in narrowing of the normal birth canal. Involvement of the thoracic region with exostoses originating from the ribs may rarely lead to spontaneous haemothorax/pneumothorax or secondary pleural irritation and effusion [6, 25, 64, 65].

### Malignant transformation

The most dangerous complication of osteochondroma is malignant transformation, which usually occurs within the cartilage cap and leads to the development of a secondary chondrosarcoma [18, 33]. Malignant transformation is estimated to occur in approximately 1% of solitary osteochondromas and 10% of HME cases [6, 8, 11]. They are usually low-grade tumours with low metastatic potential [11, 66–68], although some cases of dedifferentiation have been reported [69]. Rarely, an osteochondroma may degenerate into other malignant tumours such as osteosarcoma [6].

The average age of presentation is 31 years, with occurrences in the first decade or after the fifth decade being very rare [17]. They are usually solitary tumours, but multifocal presentations have been reported. The usual locations of secondary chondrosarcoma are rather central, notably the proximal femur, proximal humerus, scapula and pelvis [6].

Suspicion of malignant transformation should be raised in any patient with new-onset pain in the vicinity of a pre-existing osteochondroma. Imaging findings that should raise suspicion of malignancy include the growth of an osteochondroma after closure of the growth plates, irregular or lobulated margin, irregular or scattered calcifications, internal lytic areas, and erosion or destruction of adjacent bone [12, 68]. In all these cases, MRI is recommended, or CT if MRI is not possible [66].

On MRI, the main factor to be assessed to rule out malignant transformation of an osteochondroma is the thickness of the cartilaginous cap, with a thickness > 15–20 mm being suspicious of malignancy. Bernard et al [66] propose a threshold of 20 mm, with sensitivity and specificity of MRI and CT in differentiating these tumours of 100% and 98%, compared to 100% and 95%, respectively. In children, the threshold is higher (30 mm) and ultrasound assessment is accepted as an alternative [12].

**Fig. 12:**
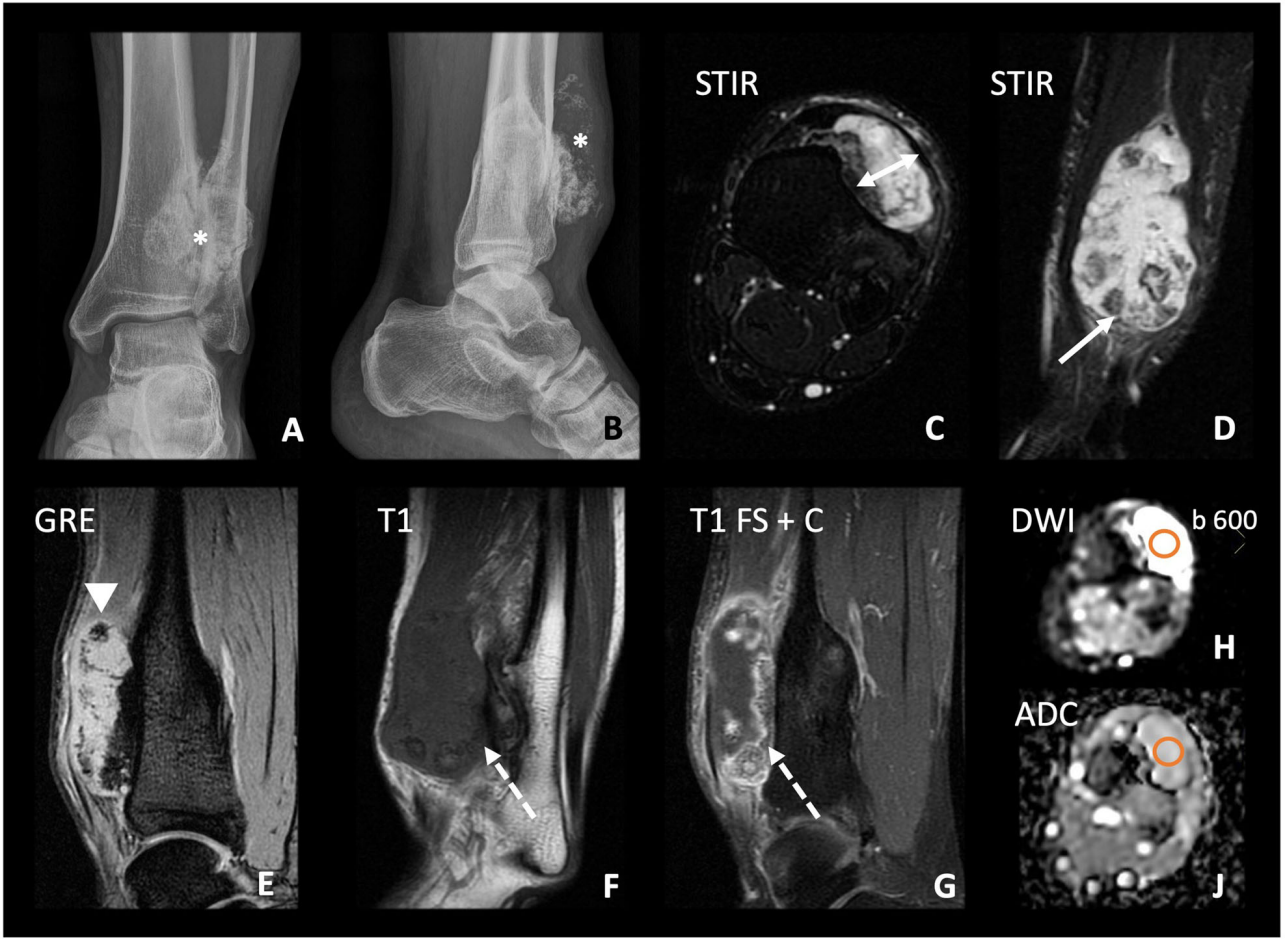
A 30-year-old man with a history of HME consulted for a painful tumour in the lower left pretibial region. AP (**A**) and lateral (**B**) radiographs of the ankle were obtained, as well as an MRI with STIR axial (**C**) and coronal (**D**) sequences, sagittal GRE (**E**), sagittal T1 (**F**), sagittal T1 FS postcontrast (**G**), DWI sequence with *b*-value of 600 (**H**) and its ADC map (**J**). A soft tissue mass is identified on the anterior aspect of the tibia (*). It has a cartilaginous cap with high SI in STIR (arrow), and intermediate SI in T1 (dashed arrow in **F**), with a thickness of up to 1.5 cm (double arrow). Calcification of the matrix with a ring-and-arc pattern is patent (*) in plain radiograph, (arrowhead in GRE). Postcontrast study shows septal enhancement (dashed arrow). In the DWI sequence, the non-mineralised part of the cartilaginous cap shows high SI, with high ADC values (2.5 × 10^−3^ in the marked ROI). Therefore, it does not restrict diffusion. The surgical specimen analysis revealed a low-grade chondrosarcoma

Other, probably less relevant, characteristics of secondary chondrosarcoma on MRI, are a lobulated appearance, with an intermediate T1W signal and an increased T2W signal due to the high water content of the cartilaginous component. The appearance is typically inhomogeneous with scattered foci of low SI due to calcification, fibrous tissue and septation [1, 5] (Fig. 12).

The use of DWI sequences has been attempted, but, to date, studies have concluded that both osteochondromas and secondary chondrosarcomas have markedly hyperintense T2W signal and a high apparent diffusion coefficient (ADC), with no significant differences between them [70]. Attempts have also been made to use enhancement patterns as a differentiator, but despite initial promising results [71], more recent studies have concluded that there are no reliable patterns to distinguish between osteochondroma and chondrosarcoma [40, 66, 67].

Bone scintigraphy cannot differentiate osteochondroma with active endochondral bone formation from lesions with malignant transformation [1]. However, [^18^F]FDG PET-CT may be useful in identifying chondrosarcomatous transformation. It has been proposed that a cut-off SUVmax value of 3.1, measured in either the stalk or the cartilaginous cap of the tumour, could differentiate benign chondral lesions from those with malignant transformation, being more useful in high-grade rather than low-grade tumours [33, 44, 45].

If malignant transformation of an osteochondroma is suspected, the patient should be referred to a specialised Sarcoma Centre for biopsy, resection and continued treatment [72]. Surgical treatment is usually performed, and the prognosis is good with a long-term survival of 75–90%. Metastases occur in 3–7% of patients, most commonly in the lungs [1, 6].

## Treatment, follow-up, and prognosis

Although there are active lines of research with promising results on RARγ agonists such as palovarotene [73, 74], there is currently no global treatment for the disease. Each lesion must be evaluated independently. Asymptomatic lesions are treated with observation only, while symptomatic lesions or lesions with suspicious imaging findings require surgical resection [1]. In asymptomatic cases without suspicious findings, intervention is not recommended, as the risk of surgery-related complications is higher than that of tumour-related complications [37, 75].

There is a local recurrence rate of 2% after excision of an osteochondroma. In all published cases there was suspicion that the initial resection may have been incomplete [5]. In children, it is preferable to postpone resection until skeletal maturity is reached, as the recurrence rate is probably higher in the immature patient [6]. If surgery cannot be postponed, the fissural plaque should be closely monitored.

For deformities secondary to HME, there are different treatments to correct them and the indication for each must be made on an individual basis. Many patients receive multiple procedures during their lifetime (2.7 on average) [50].

In the case of malignant transformation, as mentioned above, treatment is surgical. Chemotherapy and radiotherapy are only useful in dedifferentiated tumours [7]. As these are sometimes very complex procedures, 3D printing models may be useful for surgical planning [76].

Given the low local recurrence rate, the prognosis is good. Even in the case of chondrosarcoma degeneration, which is usually low grade, the survival rate 10 years after diagnosis is 83% [6].

In patients in whom a single solitary osteochondroma is detected without other associated risk data, a skeletal survey to screen for HME is not indicated, as the risk of HME is very low [5]. For the same reason, annual screening of solitary osteochondromas is also not indicated. However, when a patient is diagnosed with HME, although no follow-up plan has been shown to be superior to others, follow-up is recommended to rule out malignant transformation of any of the lesions. In our centre, we do an annual consultation and skeletal survey with conventional radiography, which is the most widespread option [8].

Since many patients with HME are diagnosed in the paediatric age, it is important to consider that they will potentially receive large doses of radiation throughout their lifetime. It is therefore very important to review the indication for each imaging test using ionising radiation that we perform in these patients. In selected paediatric cases, the follow-up may be performed with ultrasound, to avoid risks associated with excessive radiation [38].

Several studies have assessed the use of other tests, such as whole-body MRI (WB-MRI) [77, 78]. Although it has proven useful for the early detection of malignant transformation, there are still many questions about aspects such as periodicity and cost-benefit ratio.

## Conclusions

HME is an autosomal dominant disorder in which patients present with multiple metaphyseal osteochondromas affecting both flat and long bones and can be associated with various complications. Radiologists must be familiar with this condition, which can sometimes exhibit striking features from an imaging perspective, in order to make a correct diagnosis and help guide physicians and surgeons in the management of the disease, which in most cases will be conservative. However, we cannot forget that there is a risk of chondrosarcomatous transformation of some of the osteochondromas, which is rare but dangerous, so it must also be suspected and diagnosed. On the other hand, it is an open field of research, with recent advances and future projects in the fields of diagnosis with advanced MRI sequences, new therapeutic targets, preoperative planning with 3D models and new follow-up protocols using WB-MRI.

## Abbreviations

CT: Computed tomography
EXT: Exostosin
HS: Heparin sulphate
HSPGs: Heparin sulphate proteoglycans
HME: Hereditary multiple exostoses
IDH: Isocitrate dehydrogenase
MRI: Magnetic resonance imaging
SI: Signal intensity

## Declarations

### Authors’ information

Hospital Clínico San Carlos, the institution to which the authors belong, is a Spanish National Reference Center (CSUR) for sarcomas and other musculoskeletal tumours in adults and a EURACAN European Referral Center for bone sarcomas.
